# The Function of Hypoxia-Inducible Factor (HIF) Is Independent of the Endoplasmic Reticulum Protein OS-9

**DOI:** 10.1371/journal.pone.0019151

**Published:** 2011-04-29

**Authors:** Ulf Brockmeier, Corinna Platzek, Kirsten Schneider, Pauline Patak, André Bernardini, Joachim Fandrey, Eric Metzen

**Affiliations:** 1 Institute of Physiology, University of Duisburg-Essen, Essen, Germany; 2 Institute of Physiology, University of Lübeck, Lübeck, Germany; 3 Department of Neurology, University of Duisburg-Essen, Essen, Germany; University of Minnesota, United States of America

## Abstract

The protein “amplified in osteosarcoma-9” (OS-9) has been shown previously to interact with the prolyl hydroxylases PHD2 and PHD3. These enzymes initiate oxygen-dependent degradation of the α-subunit of hypoxia-inducible factor (HIF), a transcription factor that adapts cells to insufficient oxygen supply (hypoxia). A new model has been proposed where OS-9 triggers PHD dependent degradation of HIF-α. It was the aim of our study to define the molecular mode of action of OS-9 in the regulation of PHD and HIF activity. Although initial co-immunoprecipitation experiments confirmed physical interaction between OS-9 and PHD2, neither overexpression nor lentiviral inhibition of OS-9 expression affected HIF regulation. Subcellular localization experiments revealed a distinct reticular staining pattern for OS-9 while PHD2 was mainly localized in the cytoplasm. Further cell fractionation experiments and glycosylation tests indicated that OS-9 is a luminal ER protein. *In vivo* protein interaction analysis by fluorescence resonance energy transfer (FRET) showed no significant physical interaction of overexpressed PHD2-CFP and OS-9-YFP. We conclude that OS-9 plays no direct functional role in HIF degradation since physical interaction of OS-9 with oxygen sensing HIF prolyl hydroxylases cannot occur in vivo due to their different subcellular localization.

## Introduction

In yeast two hybrid screens “amplified in osteosarcoma-9” (OS-9) was identified as a protein which represses the transcription factor hypoxia-inducible factor (HIF) by activation of two enzymes that initiate oxygen-dependent degradation of HIF-α subunits [1]. Subsequently, it was reported that OS-9 is involved in endoplasmic reticulum associated degradation (ERAD) of misfolded proteins [2], [3]. It is still unclear whether these reports reflect the involvement of OS-9 in two unrelated pathways of cell metabolism, or, alternatively, suggest that OS-9 connects ERAD to hypoxic signaling. With the current study we intended to elucidate the molecular function of OS-9 in the regulation of HIF.

Molecular oxygen is the terminal electron acceptor in oxidative phosphorylation of eukaryotic cells. Coupling the breakdown of nutrients to mitochondrial respiration allows generation of much larger amounts of ATP than for example anaerobic glycolysis. Insufficient supply with oxygen, i.e. hypoxia, leads to cellular responses intended to improve oxygen delivery and to adapt metabolism to this stressful situation. A key role in this response is played by the transcription factor HIF that orchestrates the responses of the cells by activating transcription of an array of hypoxia-inducible genes [4]. HIF target genes include erythropoietin, vascular endothelial growth factor, virtually all glycolytic enzymes, membrane bound glucose transporters, and many others [5]. HIF binds to regulatory DNA regions as a heterodimer composed of an α-subunit which is quickly degraded when oxygen is abundant and a β-subunit, a nuclear protein independent of oxygen concentration. Three distinct α-subunits have been identified so far: HIF-1α and HIF-2α share similar modes of regulation and have an overlapping set of target genes while HIF-3α can act as an inhibitor of hypoxia-inducible signaling. All HIF-α subunits share the same mode of oxygen-dependent regulation which virtually eliminates HIF signaling in normoxia and strikingly induces expression of HIF target genes in hypoxia: three prolyl hydroxylases (PHD 1–3) oxidatively modify HIF-α at proline residues that are embedded in a Leu-Xaa-Xaa-Leu-Ala-**Pro** motif where Xaa depicts a non-conserved amino acid. With respect to human HIF-1α the proline residues Pro564 and Pro402 undergo hydroxylation. The next step in the degradation cascade is binding of the von-Hippel-Lindau protein (pVHL) which binds hydroxylated HIF-α selectively. Binding of pVHL is followed by ubiquitination and rapid proteasomal degradation. Despite constant production HIF-α isoforms have a half life of approximately 5 minutes in normoxia. In addition, the enzyme “factor inhibiting HIF-1” (FIH-1) hydroxylates an asparagine residue in the C-terminal transactivation domain. This reaction abrogates recruitment of transcriptional co-activators such as p300/CBP and thus represents a second switch controlling HIF-activity in an oxygen-dependent manner.

Enzymatic activity of the HIF hydroxylases is apparently tightly controlled. Molecular oxygen has two opposing effects: initially low oxygen concentrations limit enzyme turnover because the PHDs have a low affinity to oxygen as compared to collagen hydroxylases for example. Suppression of PHD activity results in HIF activation leading to enhanced transcription of the PHD2 and the PHD3 genes which have been demonstrated to be HIF targets. In turn, an increase in the expression of PHD2 and PHD3 limits HIF activity despite continuous hypoxia. In addition, PHD activity is also controlled by metabolites of the tricaboxylic acid (TCA) cycle. Succinate, lactate, pyruvate, fumarate, and oxaloacetate have been demonstrated to inhibit HIF hydroxylases although primary data have not been entirely consistent. It has been reported, however, that elevated levels of succinate and fumarate in succinate dehydrogenase or fumarate hydratase deficient tumors inhibit HIF hydroxylases and, as a consequence, activate HIF [6], [7]. Furthermore, our own data showed that nitric oxide (NO) can inhibit the HIF prolyl hydroxylases by direct inhibition of the enzyme reaction [8].

Currently, PHD2 is regarded as the dominant cellular oxygen sensor protein. This is supported by siRNA experiments in which inhibition of PHD2 led to a normoxic activation of HIF while abrogation of PHD1 or PHD3 expression did not have this effect [9]. Genetic ablation of PHD2 leads to prenatal death because of placental malformation while PHD1 and PHD3 deficient mice develop normally [10]. In adult life, PHD2 plays a prominent role in erythropoiesis [11], angiogenesis [12], matrix synthesis of human chondrocytes [13] and probably other processes which require HIF activation in response to hypoxia. In a pathophysiological situation, loss of PHD2 promotes tumor growth because angiogenesis and recruitment of bone marrow-derived cells is enhanced [14]. Interestingly, heterozygous deficiency for PHD2 in the stroma of the tumor leads to maturation of the endothelium and inhibits metastasis [15]. Clearly, understanding how PHD2 activity is regulated is of high importance in physiology and pathology.

In an early report PHD2 was purified from rabbit reticulocyte lysate. Interestingly, while the calculated molecular mass of PHD2 is approximately 46 kDa, the purified complex had an apparent molecular weight of 320–440 kDa under non-reducing conditions [16]. This finding suggests either that PHD2 is present in the complex as a multimer or that it is bound to other proteins which affect PHD2 function either as scaffold proteins or even truly regulatory subunits of the PHD complex. It was not possible so far to crystallize full length enzyme. PHD2 bearing an N-terminal deletion (PHD2_181–426_) forms a homotrimer [17] which is insufficient to explain the size of the complex. Some reports have provided evidence that the PHDs indeed have other substrates than HIF-α [18]–[20], these interactions are, however, expected to be transient in nature. Only a few proteins have been identified to interact with PHD2 without being substrates. These include the “inhibitor of growth, family member 4” (ING4, [21]), and OS-9 [1]. The repression of HIF activity by ING4 appeared to be unrelated to enzymatic PHD2 activity, but was instead dependent on the recruitment of transcriptional repressors. In contrast, the repression of HIF by OS-9 was interpreted as an activation of hydroxylase activity, and therefore seemed to be of particular interest.

The OS-9 gene was mapped to chromosome 12q13-15. Homologs of OS-9 were identified in yeast and the nematode worm *C. elegans*. The human OS-9 gene encodes a full length protein of 667 amino acid residues [22]. Shorter splice variants were also identified [23]. The function of the protein remained enigmatic until human OS-9 and its yeast homolog Yos9 were implicated in ER to Golgi transport. Notably, the subcellular localization of the protein was controversial as one group of investigators provided evidence that it is a luminal ER protein while another found OS-9 to be attached to the ER membrane but exposed to the cytoplasmic side of the membrane [24], [25]. Very recently, it was reported that OS-9 is part of the Hrd1-SEL1L multiprotein complex which is involved in retrotranslocation of misfolded proteins from the ER to the cytosol for ubiquitin dependent degradation [2]. ER associated degradation (ERAD) is of major cell physiological importance since it represents an important quality control mechanism for newly synthesized proteins. Within this process, the function of OS-9 is to bind ERAD substrates together with the ER chaperone GRP94 and to transfer the misfolded proteins to the ER membrane bound ubiquitin ligase Hrd1 [3]. The precise way in which OS-9 discriminates misfolded proteins is still unclear.

The divergent reports on OS-9 function suggested that this protein may connect HIF regulation to ER metabolism. Remarkably, effects of ER stress or intracellular calcium depletion on HIF regulation have been the subject of several publications [26]–[28]. It was the aim of our study to illuminate the molecular effects which link ER dependent metabolic steps to the regulation of the transcription factor complex HIF.

## Materials and Methods

### Antibodies and reagents

The monoclonal antibody against human HIF-1α (610959) was purchased from BD Biosciences (Heidelberg, Germany). Polyclonal antibodies against OS-9 (NB100-520) and PHD2 (NB100-137) were from Novus Biologicals (Littleton, USA). The polyclonal antibodies against actin (A2103), BiP (G6818), α-tubulin (SAB3500023) and GAPDH (G9545) were from Sigma (Munich, Germany). Polyclonal anti-lamin A (ab26300) was from Abcam (Cambridge, UK). The monoclonal, biotinylated antibody against V5-tag (MCA1360B) was purchased from AbD Serotec (Düsseldorf, Germany). The goat polyclonal, HRP-conjugated antibodies raised against rabbit (PO448) and mouse (PO447) IgG were purchased from DAKO (Hamburg, Germany). Thapsigargin, tunicamycin, puromycin, polybrene, ampicillin, kanamycin, cycloheximide and concanavalin A (C7275) were purchased from Sigma. Endo H and PNGase F were obtained from NEB (Frankfurt, Germany). Turbofect, T4 DNA ligase and all restriction enzymes were from Fermentas (St. Leon-Rot, Germany). Dimethyloxalylglycine (DMOG) was purchased from Alexis Biochemicals (Lörrach, Germany). Epoetin beta was purchased from Roche (Mannheim, Germany).

### Bacterial strains, plasmids and DNA manipulation

*E. coli* XL1-blue (Stratagene, Heidelberg) served as the host for general cloning and plasmid preparation, *E. coli* Stbl3 (Invitrogen, Darmstadt, Germany) was used for production of the lentiviral vectors pLKO.1 (8453, Addgene) and pLKO.1-shRNA-OS9 (TRCN0000156713, Sigma), the latter containing the 21 nucleotide sequence GCATCGTCTTAAACGCTACCA, corresponding to nucleotides 533–553 of OS-9 mRNA (GenBank accession number AB002806). Both *E.coli* strains were grown in LB-medium supplemented with either 100 µg/ml ampicillin or 40 µg/ml kanamycin for plasmid selection. Plasmid preparation for transient transfection into mammalian cells was performed with the High-Speed Plasmid MidiKit from Qiagen (Hilden, Germany). Plasmids encoding V5- and hexahistidine-tagged versions of PHD2 which were used in immunoprecipitation experiments were subcloned from plasmids published earlier [29]. The cDNA containing the full length OS-9 coding sequence was generously provided by Dr. Gregg Semenza (pcDNA3.1(D)/V5-His-OS-9, encoding for a C-terminal V5-His-tag and denominated pOS-9-V5 in our experiments). To generate the plasmid pEYFP-N1-OS-9 encoding OS-9 fused to the N-terminus of EYFP, a PCR was performed with forward primer 5′-ATATAAGCTTGCCACCATGGCGGCGGAAACGCTGCTG-3′ and reverse primer 5′-ATATATACCGGTGGGAAGTCAAATTCGTCCAGGTC-3′ thereby introducing restriction sites *Hin*dIII and *Age*I (underlined) for subcloning into pEYFP-N1 (Clontech, Saint-Germain-en-Laye, France). For cloning of untagged full length OS-9.1 cDNA into pcDNA3 (Invitrogen), a PCR was performed with the same forward primer and the reverse primer 5′-ATATATGCGGCCGCTCAGAAGTCAAATTCGTCCAG-3′ which contains a *Not*I site. To create expression plasmids for ECFP- and EYFP- tagged PHD2, the PHD2 coding sequence was transferred from pEGFP-N1-PHD2 [29] to pECFP-N1 and pEYFP-N1 (Clontech) by restriction digestion and ligation into the recipient vector. To generate expression plasmids for ECFP- and EYFP-fused GRP94, the cDNA of GRP94 was amplified by PCR with forward primer 5′-ATATATCTCGAGGCCACCATGAGGGCCCTGTGGGTGCTG-3′ and reverse primer 5′-ATATATGGATCCAGCAATTCATCTTTTTCAGCTGT-3′ using pCMV-SPORT6-cDNA-Grp94 (Imagenes, Berlin, Germany) as the template. The corresponding PCR fragment was cloned into the plasmids pECFP-N1 and pEYFP-N1 via the restriction sites *Bam*HI and *Xho*I (underlined). The integrity of each construct was confirmed by DNA sequencing. For transient expression of the ECFP-FKBP38 fusion protein, pECFP-C1-FKBP38 [30] was used.

### Cell culture, transfection and lentiviral transduction

U2OS, HEK 293, HEK 293T, HeLa, MCF-7, UT-7, and Hep3B cells were obtained from the American Type Culture Collection. Human foreskin fibroblasts and human umbilical vein endothelial cells (HUVEC) were provided by Promocell, Heidelberg, Germany. U2OS, HEK 293, HEK 293T, HeLa, MCF-7, Hep3B, and the fibroblasts were cultured in high glucose DMEM (Invitrogen) supplemented with 10% FBS, 2 mM glutamine and antibiotics. The non-adherent UT-7 cells were cultured in DMEM/F12 (Invitrogen) supplemented with 1 U erythropoietin/mL. HUVEC were maintained in Endothelial Cell Growth Medium (Promocell). To generate oxygen concentrations close to anoxia, cells were incubated in GasPak (BBL) anoxic jars with the BD GasPak EZ anoxic container system. Transient transfections were conducted using Turbofect (Fermentas) in a ratio 2∶1 (µl reagent/µg DNA) as recommended by the manufacturer. For production of recombinant lentivirus, 1×10^6^ HEK 293T cells were co-transfected for 4 h with 6 µg of the target vector pLKO.1-shRNA-OS-9, 4 µg of psPAX2 (12260, Addgene) and 2 µg pMD2G-VSVG (12259, Addgene). After exchange of the medium, the cells were incubated for 60 hours. The medium containing recombinant lentivirus was harvested, filtered through a 0.45 µm filter unit (Schwalbach, Germany) and stored at −80°C. Functional virus titer was calculated by infecting HEK 293T cells with limiting virus dilutions of the GFP-carrying vector pLS-CG (12161, Addgene) and subsequent quantification of GFP positive cells by fluorescence microscopy. On average, the viral titer was 1×10^6^ transduction units/ml. For transduction, 2×10^5^ U2OS cells were incubated for 16 hours with lentiviral supernatant (2×10^6^ transduction units) containing polybrene (8 µg/ml). Lentivirus infected U2OS cells were maintained in DMEM containing 1 µg/ml puromycin.

### Western blotting and Co-immunoprecipitation (Co-IP)

For Western blotting, cell extracts were prepared in RIPA lysis buffer (50 mM Tris pH 7.5, 0.1% SDS, 1% Nonidet P40, 0.5% sodium deoxycholate, 2 mM EDTA, 150 mM NaCl) containing protease inhibitor cocktail (Roche, Mannheim Germany). Protein samples were separated on 7.5% reducing SDS gels and electroblotted onto PVDF membranes. After the transfer, blocking of unspecific binding sites was achieved by incubation in TBST (50 mM Tris/HCl, 150 mM NaCl, 0.5% Tween 20, pH 7.2) containing 5% skimmed milk. For the incubation steps with primary and HRP-conjugated secondary antibodies, antibody concentrations were used as recommended by the manufacturer. Detection was performed with the *ECL* kit (GE Healthcare, Munich, Germany) using the FX7 chemoluminescence documentation system (Peqlab, Erlangen, Germany). For Co-IP, U2OS cells were co-transfected with the expression vectors pOS-9-V5 and pPHD2-His. After 48 h, cells were lysed in NP40 lysis buffer (1% Nonidet P40, 150 mM NaCl, 2 mM EDTA, 10 mM sodium phosphate, pH 7.2) and directly subjected to immunoisolation with a biotinylated antibody against V5-tag for 2 h at 4°C. Immune complexes were recovered by incubation with streptavidin agarose (Invitrogen) for 1 h at 4°C. The beads were washed 3 times with NP40 buffer, resuspended in sample buffer and boiled for 5 min at 95°C. After removal of the beads by centrifugation, the supernatant was analyzed by reducing SDS-PAGE and immunoblotting.

### Subcellular fractionation

For isolation of the cytoplasm, the cells were trypsinized and washed in sucrose buffer (5 mM sucrose, 1 mM Hepes, pH 7.4), resuspended in sucrose buffer supplemented with 50 µg/mL digitonin and incubated on ice for 2 min. Following centrifugation (1 min, 800 g, 4°C), the supernatant was removed and designated the cytoplasmic fraction. The pellet was washed several times with digitonin-free sucrose buffer and was used as the organelle fraction which was solubilized in RIPA buffer. Both fractions were subjected to SDS-PAGE and immunoblotting. For isolation of the nuclear fraction, cells were lysed using the NE-PER Nuclear and Cytoplasmic Extraction Kit (Thermo Scientific, Rockford, USA). For isolation of the endomembranes, cells were trypsinized, washed in PBS and resuspended in hypotonic buffer HB (10 mM Hepes, 10 mM MgCl_2_, 42 mM KCl, protease inhibitors, pH 7.4) and incubated on ice for 5 min. Cells were broken with 3–5 passages through a 30½G needle and centrifuged (600 g, 10 min, 4°C) to separate the postnuclear supernatant from the pellet containing the crude-nuclei-fraction. The postnuclear supernatant was subjected to an ultracentrifugation step (100000 g, 90 min, 4°C) to separate endomembranes (pellet) from cytosol (supernatant). The pellet was washed three times with HB, resuspended in 1 M KCl and incubated for 20 min on ice. The supernatant was isolated and designated the wash fraction. After extensive washing in HB, the pellet was resuspended in RIPA buffer. Endomembranes, cytosolic fraction and wash fraction were subjected to SDS-PAGE and immunoblotting.

### Fluorescence resonance energy transfer (FRET)

FRET measurements were performed as described previously [31]. Briefly, HEK293 cells were transfected transiently for 48 h with the appropriate ECFP- and EYFP-fusion plasmids. The cell culture dishes were subjected to in vivo imaging on the microscope stage. An inverted microscope (Axiovert 200 M, Carl Zeiss Microimaging GmbH, Germany) coupled to a Nipkow Disc system QLC100 (Visitech International Ltd., UK) was used for confocal microscopy. Two diode-pumped solid state lasers provided excitation wavelengths of 444 nm (CFP) and of 532 nm (YFP). The emitted light was detected after passage through the Nipkow disc unit with a CCD camera (Orca ERG, Hamamatsu, Japan). To avoid bleaching, a Multispec MicroimagerDualView beam splitter (Optical Insights, Tucson, AZ) was installed for simultaneous detection of two emission wavelengths on the CCD chip. Assuming that the value of non-radiation relaxation processes during/after FRET is negligible we calculated FRET efficiency for each pixel in the images by the following formula:
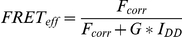
where
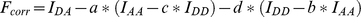
In this formula, the factor G relates the FRET efficiency to the acceptor photo bleaching method and therefore renders the resulting FRET efficiency independent of the underlying imaging setup as described earlier [32].

I denotes the pixel-by-pixel intensity as captured by the camera. Indices of I represent excitation source and detection channel, respectively (I_DA_ = acceptor emission intensity during donor excitation; I_AA_ = acceptor emission intensity during acceptor excitation; I_DD_ = donor emission intensity during donor excitation). Factors a–d are bleedthrough and crosstalk calibration factors. All factors were determined as described by Zal and Gascoigne [32]. To obtain various acceptor to donor (A/D) ratios, transfections were conducted with different DNA ratios of acceptor/donor plasmids (1∶1 up to 5∶1) to achieve FRET efficiencies over this range of values. The relationship between FRET efficiency and A/D ratio was modeled with a simple 1∶1 ligand binding model as described before [33], [34]. For statistical analysis of the FRET results the software programs “ANUFIS” (Automated and Uncomplicated FRET Imaging Solution) [35] and SigmaPlot 2004 (SYSTAT, Chicago, USA) were used. For interpretation of the FRET measurements, only data from A/D ratios higher than 1 of the resulting regression functions were considered, since lower A/D ratios favour the noise level over the signal and are therefore highly prone to measurement errors. Furthermore, at high A/D ratios, the probability for donors which are not paired with acceptors tends towards zero. In such cases FRET efficiency converges to its theoretical value for a one-dimensional scenario as characterized by the Förster equations.

## Results

### OS-9 interacts with PHD2 in vitro but not with HIF-1α

**Figure 1:**
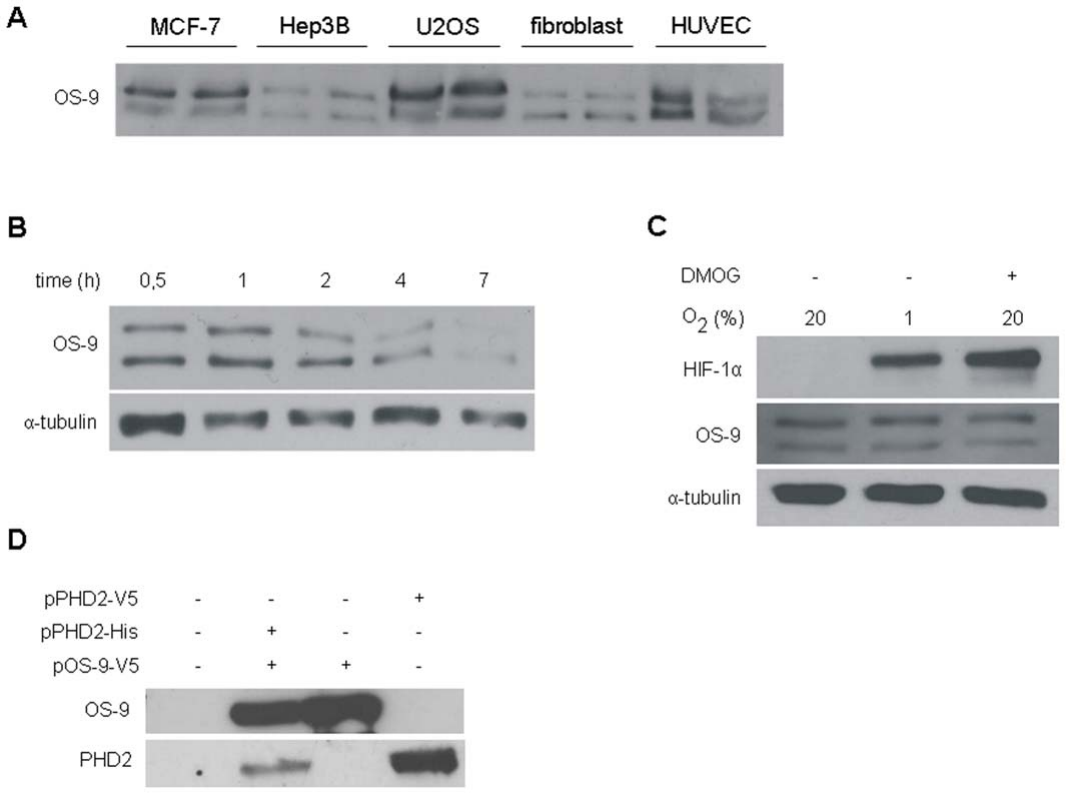
Initial characterization of the OS-9 protein. (A) OS-9 expression in various human cell lines. Equal protein amounts of total cell lysates were used for SDS-PAGE and subsequent Western blotting. For each cell line, two independent samples are shown. Endogenous OS-9 was detected with a polyclonal antibody raised against a peptide corresponding to amino acids 600–667 of isoform 1 of OS-9. (B) Protein stability assay of endogenous OS-9. U2OS cells were treated with the translational inhibitor cycloheximide (100 µM). At indicated time points, whole cell lysates were analysed by immunoblotting. (C) Effect of hypoxia on OS-9 expression. For hypoxia, UT-7 cells were exposed to 1% O_2_ for 24 h prior to Western blot analysis. To determine any influence of HIF-1α on OS-9 expression under normoxia, cells were incubated with the prolyl hydroxylase inhibitor DMOG (0.5 mM) for 24 h. (D) Protein interaction between OS-9 and PHD2 *in vitro*. For co-immunoprecipitation, U2OS cells were transiently co-transfected with the plasmids pOS-9-V5 and pPHD2-His, lysed in NP40 buffer, and subjected to immunoisolation with anti-V5 antibody recognizing OS-9 by its V5-tag. OS-9 and its associated proteins were separated by SDS-PAGE and analyzed by Western blot (lane 2). As controls, samples of untransfected (lane 1) cells or cells transfected with a single plasmid (lanes 3–4) were loaded. Representative Western blots are shown for each subfigure.

Data published previously showed a strong interaction of OS-9 with HIF-1α and PHD-2 and suggested a direct role of OS-9 in oxygen-dependent degradation of HIF-1α in the cytoplasm [1]. However, subsequent studies performed by several independent research teams revealed that OS-9 also plays an important part in the ER degradation pathway of misfolded proteins (ERAD). The initial focus of this study was to describe the role of OS-9 in the regulation of O_2_ homeostasis more precisely. In a first set of experiments we established OS-9 Western blotting using a commercial antibody raised against OS-9: Two of the 3 splice variants described previously [36] were detectable in various transformed and untransformed cell lines (Fig. 1A). Alternative splicing was confirmed by RT-PCR. Sequencing of RT-PCR products from U2OS total RNA identified version 1 which contains all exons and version 2 which lacks exon 13. Both splice variants had a half-life of approximately 4 hours (Fig. 1B) and their expression levels were not influenced by hypoxia or DMOG (Fig. 1C). Next, we investigated the binding ability of OS-9 to PHD2 in human cells. After co-transfection of U2OS cells with expression vectors encoding hexahistidine tagged PHD2 and V5-tagged OS-9, the cell lysates were incubated with a biotinylated anti-V5 antibody. Analysis of the immunoprecipitated fraction by Western blotting indeed revealed protein interaction between PHD-2 and OS-9 (Fig. 1D, lane 2). Interestingly, we were not able to detect any endogenous PHD-2 or OS-9 after immunoprecipitation when the cells were transfected with a single plasmid encoding V5-tagged OS-9 or PHD2, respectively (Fig. 1D, lane 3 and 4). To demonstrate protein binding of OS-9 and HIF-1α as well, we performed another co-immunoprecipitation experiment using expression vectors for V5-tagged OS-9 and HIF-1α. However, in our hands no co-immunoprecipitation of HIF-1α was detectable (data not shown).

### Oxygen-dependent regulation of HIF-1α is independent of OS-9

**Figure 2:**
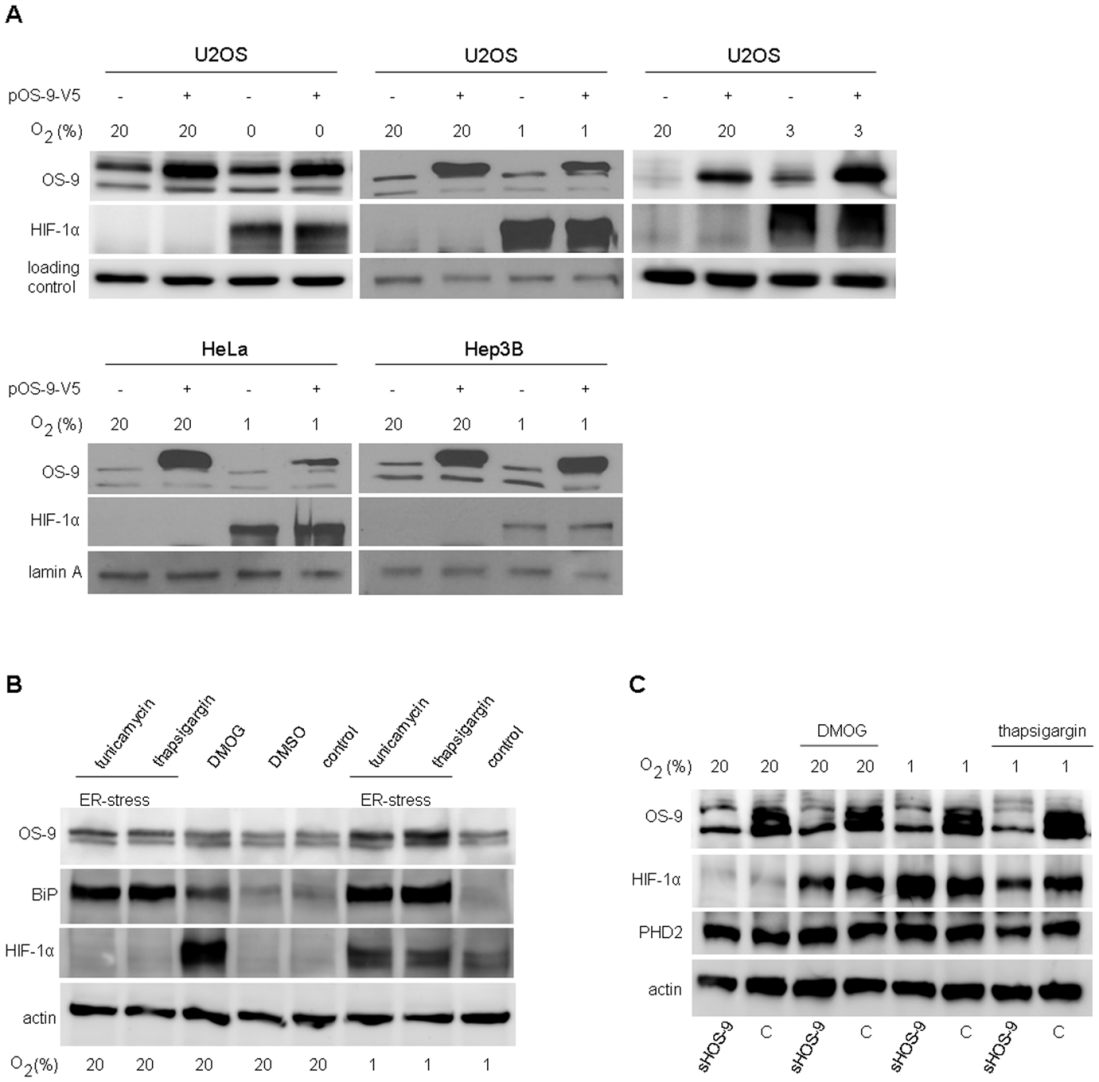
OS-9 shows no effect on regulation of HIF-1α. Total cell lysates were used for SDS-PAGE and subsequent Western blotting. To generate nearly anoxic conditions, cells were exposed to an oxygen consuming chemical system or to 1% or 3% O_2_ for 4 h to generate hypoxia. (A) U2OS, HeLa and Hep3B cells were transiently transfected with the plasmid pOS-9-V5 48 h prior to the experiment. Lamin A and actin were used as loading controls. (B) U2OS cells were subjected to ER stress by incubation either with tunicamycin (1 µg/ml) or thapsigargin (0.5 µg/ml) for 20 h. To detect HIF-1α under normoxia, cells were treated with DMOG (1 mM) for 4 h. A sample of DMSO-only treated cells was loaded to exclude unspecific side effects of the solvent. (C) U2OS cells were transduced with lentiviral construct pLKO.1-shRNA-OS-9 (shOS-9) mediating a stable knockdown of OS-9 expression. Control cells (c) were transduced with plasmid pLKO.1-puro. Representative Western blots are shown for each subfigure.

To examine whether the protein interaction between OS-9 and PHD2 has relevance for the regulation of HIF-1α in living cells, we performed gain- and loss-of-function experiments. Initially, we overexpressed OS-9 transiently in U2OS cells and analyzed the HIF-1α level under normoxic and hypoxic conditions by immunoblotting (Fig. 2A). In normoxia, HIF-1α was not detectable due to complete oxygen-dependent degradation (Fig. 2A, upper panel). Notably, we were unable to demonstrate a significant difference in the level of HIF-1α neither when the oxygen concentration was close to anoxia nor under hypoxia (1% and 3% O_2_). A cell type specific effect seemed unlikely since this experiment showed the same outcome in two other cell lines, HeLA and Hep3B (Fig. 2A, lower panel). Next, we treated U2OS cells with tunicamycin and thapsigargin. Both reagents induce upregulation of ER chaperones by ER stress. We tested the cell lysates for BiP, OS-9 and HIF-1α expression (Fig. 2B). As expected, the expression of one of the major ER chaperones, BiP, was induced under ER stress in normoxia and hypoxia (Fig. 2B, lanes 1–2, 6–7). Since the level of OS-9 was elevated after ER stress as well, this indicated a link between OS-9 and the ER. Remarkably, in hypoxia HIF-1α induction was enhanced in response to ER stress while the OS-9 levels were elevated as well (Fig. 2B, lanes 6–8). In addition, we treated the cells under normoxia with the prolyl hydroxylase inhibitor DMOG to monitor whether stabilized HIF-1α shows an effect on the expression level of OS-9 (Fig. 2B, lane 3). However, and in line with Fig. 1C, this was clearly not the case which implies that OS-9 is not a target gene of HIF-1α. Finally, we tested whether the loss of function of OS-9 influences cellular HIF-1α protein. To suppress OS-9 expression, lentiviral particles which induce production of a small hairpin RNA against OS-9 were used to infect U2OS cells. The transduced cell line showed a significant OS-9 knockdown in normoxia and hypoxia when compared to the control cells (Fig. 2C). In line with the results of the gain-of-function experiments, inhibition of OS-9 did not have a detectable influence on HIF-1α expression levels, neither in normoxia nor in hypoxia with or without ER stress. Noteworthy, under normoxia, HIF-1α remained undetectable which suggests that degradation by PHD2 was not impaired in the OS-9 knockdown cell line (Fig. 2C, lane 1 and 2). Additional experiments were conducted in U2OS cells with a transient knockdown of OS-9 by transfection of the cells with specific siRNA. The efficiency of OS-9 knockdown by siRNA transfection was variable. However, and in line with our results for the lentivirally mediated OS-9 knockdown, HIF-1α expression was apparently not correlated to OS-9 protein levels (data not shown). The siRNA data also exclude unspecific effects of the lentiviral infection on the outcome of the experiments.

### Discrepant localization of OS-9 and PHD2 in the cell

**Figure 3:**
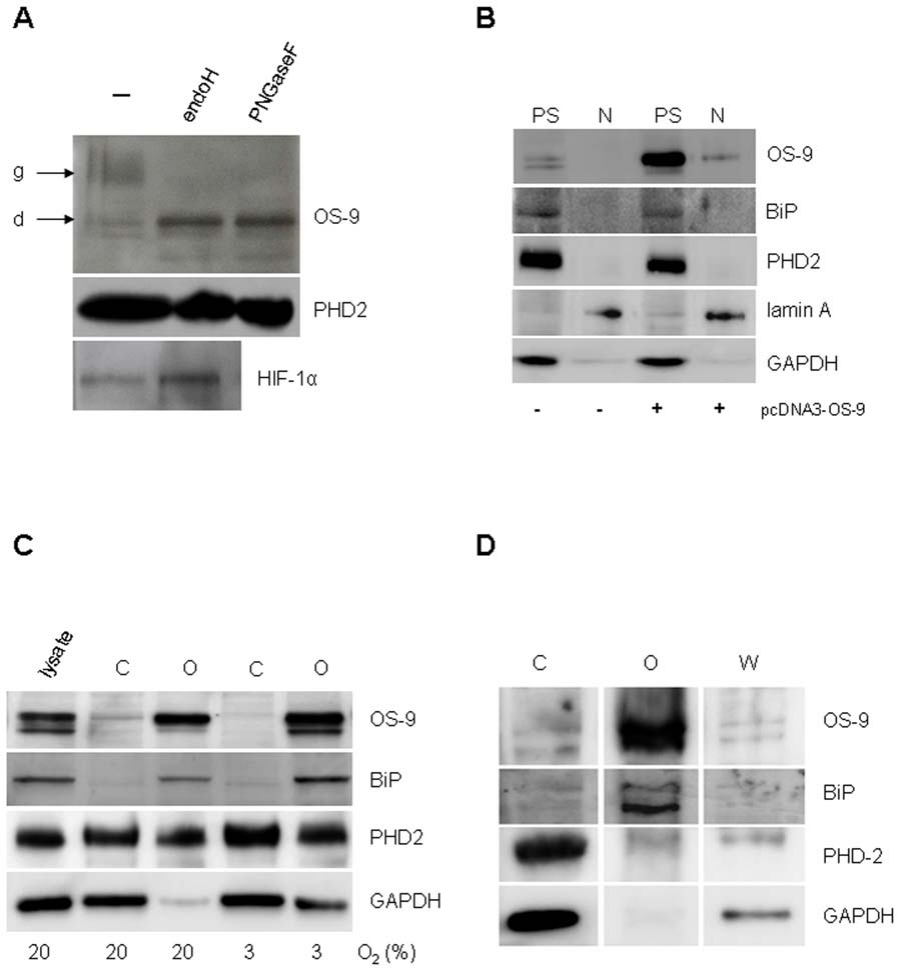
Cellular localization of OS-9 and PHD2. (A) A lectin gel-shift assay was conducted to test for glycosylated proteins. Total cell lysates of U2OS cells were incubated in the presence or absence of the endoglycosidases EndoH and PNGaseF for 6 h at 37°C. Digest products were separated on a reducing 10% SDS-PAGE gel which contained concanavalin A co-polymerized in the top layer of the separating gel to retard mobility of glycosylated proteins [56]. Glycosylated OS-9 is indicated as ‘g’, deglycosylated OS-9 as ‘d’. (B) Detection of OS-9 and PHD2 in the nuclear fraction. HEK293 cells with and without transfection of the plasmid pcDNA3-OS-9 were separated into nuclear fraction (N) and postnuclear supernatant (PS), the latter containing cytoplasm and organelles. Western blot analysis included BiP, GAPDH and lamin A as typical marker proteins for the ER, the cytoplasm and the nucleus, respectively. (C) Detection of OS-9 and PHD2 in the cytoplasm. HEK293 cells were co-transfected with pcDNA3-OS-9 and pPHD2-V5. For hypoxia, cells were exposed to 3% O_2_ for 4 h. Cells were treated with 50 µg/ml digitonin and centrifuged to obtain a cytoplasmic (C) and an organelle fraction (O) and subjected to immunoblotting. (D) Isolation of cellular endomembranes. HEK293 cells were lysed mechanically by several passages through a 30½G needle. The postnuclear supernatant was processed further by ultra-centrifugation to separate the organelles (O) from the cytosol (C). High salt treatment (1 M KCl) of the organelle fraction produced a wash fraction (W) that contained dissociated peripheral membrane proteins. For immunoblot analysis of subcellular fractionations, cell aliquots were normalized for cell number prior to loading (B–D). Representative Western blots are shown for each subfigure.

Although a robust protein interaction between OS-9 and PHD2 was confirmed in our co-IP experiments, OS-9 did not have any physiological impact on the oxygen-dependent regulation of HIF-1α in living cells as reported previously. In order to clarify this apparent discrepancy, we decided to investigate whether both proteins co-localize under physiological conditions, i.e. when cell compartments are fully intact. OS-9 shows an N-terminal leader peptide but lacks a typical C-terminal ER-retention signal (KDEL) suggesting at least transient localization in the ER, the cell compartment where glycosylation and folding of most export proteins take place. Since OS-9, PHD2 and HIF-1α all possess potential glycosylation sites (Asp-X-Ser/Thr), we tested them for glycosylation as an index for presence in the ER. Thus, cell lysates of U2OS cells were incubated with two different endoglycosidases and subsequently analyzed by Western blot (Fig. 3A). EndoH cleaves off high mannose carbohydrates which is typical for glycoproteins of the ER, whereas PNGaseF deglycosylates also complex oligosaccharides from glycoproteins travelling through the Golgi. In contrast to PHD2 and HIF-1α, a pronounced mobility shift identified OS-9 as a glycoprotein when treated with either endoglycosidase (Fig. 3A, compare lane 1 with lane 2 and 3). A minor fraction of OS-9 with a lower molecular weight was visible, indicating a second, unglycosylated form of OS-9 (Fig. 3A, lane 1). However, this naturally non-glycosylated OS-9 has the same mobility as the Endo H- or PNGase-cleaved material. Consequently, it has fully undergone signal peptide cleavage and translocation into the ER. Hence, OS-9 is presumably a soluble glycoprotein located in the ER-lumen since sequence analysis did not predict a membrane anchor. Next, various methods of cell fractionation were conducted to elucidate the exact cellular localization of OS-9 and PHD2. First, the nuclear fraction of HEK293 cells was tested for OS-9 and PHD2 as shown in Fig. 3B. Under normoxia, endogenous OS-9 and PHD2 were exclusively detected in the non-nuclear fraction (cytoplasm and organelles) but not in the nucleus. Of note, when OS-9 was overexpressed, a small proportion was present in the nuclear fraction (Fig. 3B, lanes 3 and 4). Detection of the ER resident protein BiP, the nuclear protein lamin A and the cytoplasmic protein GAPDH confirmed integrity of the cell compartments after fractionation. Subsequently, the cytoplasm was tested for OS-9 and PHD2. Treatment of the cells with the mild non-ionic detergent digitonin allows exclusively an efflux of soluble cytoplasmic proteins through the formation of pores in the cell membrane. OS-9 was detectable in the organelle fraction, whereas PHD2 was localized mainly in the cytoplasm (Fig. 3C). Nevertheless, there was a minor amount of PHD2 found in the organelle fraction which is in line with previously published data showing that PHD2 is to some part non-covalently bound to the peptidyl-prolyl cis/trans isomerase FKBP38, a transmembrane protein anchored to the cytoplasmic side of the ER membrane [30]. The control protein GAPDH is typically found in the cytoplasm (Fig. 3C, lane 2 and 4), but cell stress (here hypoxia) is known to trigger translocation into the nucleus [37] (Fig. 3C, lanes 3 and 5). However, since we also found PHD2 in the organelle fraction, we decided to analyze its localization with a different method in which the organelle fraction is isolated after mechanical cell disruption and subsequent ultra-centrifugation. As expected, OS-9 protein was detectable in the organelle fraction, whereas PHD2 was localized predominantly in the cytoplasm (Fig. 3D). An additional washing step of the organelle fraction with 1 M KCl did not reveal OS-9 protein loosely bound to the cytoplasmic side of the ER membrane as was the case for our cytoplasmic marker GAPDH (Fig. 3D). We noticed a faint amount of OS-9 visible in both, the cytoplasmic and the wash fraction which could argue for a minimal cytosolic fraction of OS-9. However, since the ER luminal protein BiP displayed a very similar distribution pattern after cell fractionation, it seemed more likely that these signals represent inevitable contaminations.

### OS-9 and PHD2 do not show protein-protein interaction in vivo

**Figure 4:**
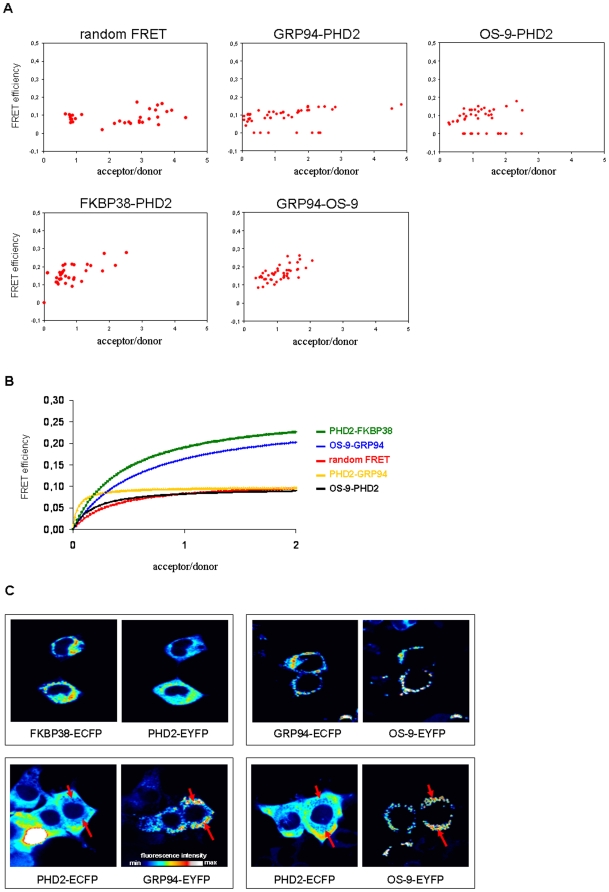
FRET analysis of protein interaction between OS-9 and PHD2. HEK293 cells were transiently co-transfected with the appropriate ECFP- and EYFP fusion plasmids. After 48 h, live-cell imaging was conducted by confocal microscopy. (A) FRET efficiencies for each ECFP- and EYFP fusion protein pair were calculated from 25–45 randomly selected cells which displayed various fluorescent acceptor/donor ratios. (B) Scatter plots were fit to a single-site binding model. FRET efficiency E is defined as the percentage of transferred energy relative to the energy absorbed by the donor. Additional details are given under ‘Materials and Methods’. For random FRET, cells were co-transfected with plasmids pECFP-N1 and pEYFP-N1 expressing non-fused ECFP and EYFP. (C) Fluorescent images of co-transfected cells were acquired separately through CFP- and YFP-filter channels using a 40× objective. The fluorescence intensity is visualized in false colors on a color bar from low (blue) to high intensity (white). Marked by red arrows are cellular areas of very low fluorescence intensity in the CFP-filter channel and of high fluorescence intensity in the YFP-filter channel. Each experiment was performed at least three times, representative data are shown.

In addition to our in vitro approach, we performed protein interaction studies of OS-9 and PHD2 using FRET (fluorescence resonance energy transfer) analysis. Due to the dependence of FRET on proximity, it provides an excellent assay for monitoring the interaction of two proteins in living and intact cells. The transfer of energy from the donor PHD2-ECFP to the acceptor OS-9-EYFP fusion protein can only occur when the two molecules are in close proximity (<10 nm), consistent with being in molecular contact [38]. Consequently, various ECFP- and EYFP-fused OS-9, PHD2, GRP94 and FKBP38 proteins were expressed in U2OS cells and used in different combinations for FRET measurements (Fig. 4A). Initially, we determined random FRET as a control which is derived from random collisions between EYFP and ECFP only (not fused to any other protein). This curve depicts a maximum FRET efficiency of approximately 10% (Fig. 4B). However, as one of the proteins under scrutiny presumably localizes to the lumen of the ER we reasoned that ECFP/EYFP interaction is not a perfect control. Therefore, we used PHD2/FKBP38 interaction and OS-9/GRP94 interaction as positive controls. In contrast, PHD2/GRP94 interaction was regarded as a negative control because GRP94 is well-documented to be a luminal ER protein while PHD2 is predominantly cytoplasmic. Only at very low acceptor to donor ratios the signal was marginally stronger than EYFP/ECFP random FRET (Fig. 4B). These experiments demonstrated that FRET levels did not exceed background when PHD2 interaction with OS-9 was tested (<10% maximum FRET efficiency) suggesting no specific interaction between these two molecules. However, clearly positive FRET signals were found when OS-9 and GRP94 or when PHD2 and FKBP38 (>20% maximum FRET efficiency) were expressed (Fig. 4B), confirming interactions of these proteins as published previously [3], [30]. As depicted in Fig. 4C, the images taken from the CFP- and YFP-emission channels revealed clearly a cytoplasmic distribution of PHD2 in contrast to OS-9 and GRP94 which are both localized in a perinuclear compartment. Collectively, these data suggest that under physiological conditions, OS-9 and PHD2 are unable to interact in vivo due to their localization in different compartments of the cell, OS-9 in the ER lumen and PHD2 in the cytoplasm.

## Discussion

The oxygen sensing prolyl hydroxylases PHD2 and PHD3 play a central role in the regulation of cell metabolism because they constantly initiate degradation of HIF-α as long as oxygen is present. The reduction of enzyme turnover by a lack of oxygen consequently leads to the activation of HIF which in turn stimulates transcription of approximately 2% of all active genes for example in endothelial cells [39]. Importantly, several situations have been described in which HIF is active despite normal levels of oxygen. This normoxic activation may be caused by enhanced transcription of the HIF-1α gene itself [40], [41] or by a defect of HIF-α degradation, e.g. when cells are deficient in functional pVHL [42] or when elevated levels of TCA cycle intermediates inhibit the HIF hydroxylases [43]. Remarkably, previous studies suggested that calcium depletion and ER stress can also affect hypoxic signaling either via interference with the regulation of HIF [26]–[28] or via HIF-independent pathways [44]. The protein OS-9 was reported to be involved in both, the activity regulation of HIF and ER associated degradation, and thus promised to be a molecular link between these processes.

The ER represents the site of synthesis for most glycoproteins destined for secretion or for integration into the plasma membrane [45]. It also functions as the main Ca^2+^ repository of the cell which is pivotal for nascent proteins to reach their native conformation. A Ca^2+^ reduction in this organelle results in impaired protein folding and ER stress [46]. Non-native substrates are held back in the ER and finally selected for a process termed ERAD which leads to retrotranslocation into the cytosol, ubiquitination and degradation by the 26S proteasome [47]. Recent reports suggest that mannose trimming of misfolded glycoproteins by ER mannosidases in conjunction with EDEM1-3 generates an oligosaccharide form that serves as a degradation signal [48]–[50]. This promotes interaction with the newly characterized ERAD component OS-9 which facilitates its targeting to the retrotranslocation channel, although the detailed molecular mechanism behind this process is still not fully understood [2], [3], [51].

The initial study which implicated OS-9 in PHD regulation was based on a yeast two hybrid screen which identified OS-9 as a protein interacting with HIF. Within this report physical interaction of over-expressed OS-9 with HIF-1α and PHD2 was detected in cultured cells by co-immunopreciptitation. Additionally, cell fractionation experiments were performed in a way which discriminates between nucleus and cytoplasm. In one study published recently interaction of OS-9 with HIF could not be confirmed [52] which is in line with our data. Using a new, commercially available antibody raised against endogenous OS-9 we first demonstrated that OS-9 is expressed in transformed as well as untransformed cell lines that OS-9 has a protein half-life of approximately 4 hours and that OS-9 expression is not regulated by hypoxia. Of note, several components of the HIF system are hypoxia-inducible such as PHD2 and thus modify activity of the system in a feedback loop. However, the antibody was not sensitive enough to be used in immunofluorescence experiments. Although our experiments also showed binding of OS-9 to PHD2 *in vitro* we were unable to demonstrate effects of OS-9 manipulation on HIF regulation. We over-expressed OS-9 or attempted to reduce OS-9 levels either by transient transfection of short interfering RNA or short hairpin vectors. Interestingly, transient transfection methods had no effect or led to a variable reduction of cellular OS-9 levels. Of note, however, when incubated in hypoxia siRNA transfected cells did not display a significant correlation between the reduction of OS-9 levels and the hypoxic induction of HIF. We achieved a better knockdown of OS-9 when we constructed recombinant lentiviral particles to deliver short hairpin vectors to cultured cells. These vectors integrate into the host genome and lead to a stable reduction of target gene expression. In our hands, the OS-9 knockdown was stable for three weeks at least. Disappointingly, despite high efficiency of the OS-9 knockdown the cells responded normally to hypoxia in terms of HIF regulation.

We noted that subcellular localization of OS-9 was discussed controversially in recent reports. While one group found OS-9 attached to the cytoplasmic side of the ER membrane [25] other groups reported that OS-9 is a luminal ER protein [53]. Of note, in one case a leakage of OS-9 out of the ER lumen was documented, but only when OS-9 was substantially overexpressed [3]. Cell fractionation experiments cannot provide unequivocal evidence that all OS-9, endogenous as well as overexpressed, localizes to the lumen of the ER. On some occasions we detected OS-9 in the nuclear compartment. This result is, however, not surprising since the ER is bound tightly to the nuclear envelope and there may even be some exchange between nuclear and ER proteins. Clearly, OS-9 best co-localized to BiP which is a marker for luminal ER proteins. We also performed high salt washes of endosome preparations to exclude binding of OS-9 to the cytoplasmic side of the ER. This procedure did not lead to a release of OS-9 from the endosomes. Because OS-9 does not possess a transmembrane domain these experiments strongly argue in favor of an indeed luminal localization.

Nevertheless we intended to analyze in vivo protein interaction between OS-9 and PHD2 to prove or disprove binding in a situation when cellular membranes and barriers are intact. To this end we used fluorescence resonance energy transfer (FRET) which we have used previously to analyze binding of HIF-1α to HIF-1β [54] and to prove interaction of PHD2 with the cis/trans prolyl isomerase FKBP38 [30]. We fused the PHD2 coding sequence to ECFP and the OS-9 coding sequence to EYFP. In theory, a positive FRET signal requires physical contact of the fusion proteins, because only in this case energy can be transferred from the donor protein ECFP to the acceptor EYFP. In reality, however, in addition to random collisions of ECFP and EYFP which both localize to the same subcellular compartments, there is a weak but detectable overlap between the emission spectra of ECFP and EYFP which we tried to correct mathematically. At very low acceptor to donor ratios an arguable, low level of FRET signal was detectable in OS-9/PHD2 interaction experiments. Finally, we used organelle specific FRET between OS-9 and GRP94 as a positive control. GRP94 is a well-characterized luminal ER protein and was reported previously to interact with OS-9 [3]. Interaction experiments of OS-9 with GRP94 gave a strong FRET signal. As reported previously we also confirmed PHD2 interaction with FKBP38 as an example of a protein which robustly interacts with PHD2 in vivo. We then tested PHD2 interaction with GRP94 to define the background level of fluorescent emissions of the acceptor protein EYFP in our FRET system. In this experiment, we also measured low level FRET signals at very low acceptor to donor ratios. Importantly, OS-9/GRP94 FRET signals exceeded OS-9/PHD2 FRET signals. Therefore our results demonstrate that OS-9-EYFP interaction with PHD2-ECFP does not exceed background levels and make physical interaction of OS-9 with PHD2 in living cells highly unlikely.

It remains unclear what the molecular link between ER stress and HIF-dependent signaling is. Indeed we have repeatedly noticed an augmentation of HIF activation by tunicamycin and thapsigargin. These substances induce ER stress in different ways: tunicamycin blocks N-linked glycosylation of nascent proteins while thapsigargin inhibits the ER Ca^2+^ pumps [55]. In our hands both agents induce BiP and OS-9 which supports recent reports that demonstrate OS-9 induction by ER stress [53]. Simultaneous induction of HIF and OS-9, however, strongly argues against activation of HIF hydroxylases by direct physical interaction with OS-9. Therefore the molecular link between hypoxic and ER stress signaling is still elusive.
